# Beer

**Published:** 1886-02

**Authors:** 


### BEER.

For some years a decided inclination has been apparent all over the country to give up the use of whiskey and other strong alcohols, using as a substitute beer and other compounds. This is evidently founded on the idea that beer is not harmful, and contains a large amount of nutriment; also that bitters may have some medical quality which will neutralize the alcohol which it conceals, etc. These theories are without confirmation in the observation of physicians. The use of beer is found to produce a species of degeneration of all the organs; profound and deceptive fatty deposits, diminished circulation, condition of congestion, and perversion of functional activities, local inflammations of both the liver and kidneys, are constantly present. Intellectually a stupor amounting almost to paralysis arrests the reason changing all the higher faculties into a mere animalism, sensual, selfish, sluggish, varied only with paroxysms of anger that are senseless and brutal. In appearance the the beer drinker may be the picture of health, but in reality he is most incapable of resisting disease. A slight injury, a severe cold, or a shock to the body or mind will commonly provoke acute disease ending fatally.

Compared with inebriates who use different kinds of alcohol, he is more incurable and more generally diseased. The constant use of beer every day gives the system no recuperation, but steadily lowers the vital forces. It is our observation that beer drinking in this country produces the very lowest kind of inebriety, closely allied to criminal insanity. The most dangerous class of ruffians in our cities are beer drinkers.—*Scientific American.*

